# In Vitro Downregulation of Matrix Metalloproteinase-9 in Rat Glial Cells by CCR5 Antagonist Maraviroc: Therapeutic Implication for HIV Brain Infection

**DOI:** 10.1371/journal.pone.0028499

**Published:** 2011-12-08

**Authors:** Pasqua Gramegna, Tiziana Latronico, Maria Teresa Branà, Gaetano Di Bari, Fabio Mengoni, Valeria Belvisi, Maria T. Mascellino, Miriam Lichtner, Vincenzo Vullo, Claudio M. Mastroianni, Grazia M. Liuzzi

**Affiliations:** 1 Department of Biochemistry and Molecular Biology, University of Bari, Bari, Italy; 2 Department of Public Health and Infectious Diseases, “Sapienza” University, Rome, Italy; 3 Fondazione Eleonora Lorillard Spencer Cenci, Infectious Diseases Unit, “Sapienza” University, Polo Pontino, Latina, Italy; Mayo Clinic, United States of America

## Abstract

**Background:**

Matrix metalloproteinases (MMPs) released by glial cells are important mediators of neuroinflammation and neurologic damage in HIV infection. The use of antiretroviral drugs able to combat the detrimental effect of chronic inflammation and target the exaggerated MMP activity might represent an attractive therapeutic challenge. Recent studies suggest that CCR5 antagonist maraviroc (MVC) exerts immunomodulant and anti-inflammatory activity beyond its anti-HIV properties. We investigated the in vitro effect of MVC on the activity of MMPs in astrocyte and microglia cultures.

**Methodology/Principal Findings:**

Primary cultures of rat astrocytes and microglia were activated by exposure to phorbol myristate acetate (PMA) or lypopolysaccharide (LPS) and treated in vitro with MVC. Culture supernatants were subjected to gelatin zymography and quantitative determination of MMP-9 and MMP-2 was done by computerized scanning densitometry. MMP-9 levels were significantly elevated in culture supernatants from both LPS- and PMA-activated astrocytes and microglia in comparison to controls. The treatment with MVC significantly inhibited in a dose-dependent manner the levels and expression of MMP-9 in PMA-activated astrocytes (p<0,05) and, to a lesser extent, in PMA-activated microglia. By contrast, levels of MMP-2 did not significantly change, although a tendency to decrease was seen in PMA-activated astrocytes after treatment with MVC. The inhibition of levels and expression of MMP-9 in PMA-activated glial cells did not depend on cytotoxic effects of MVC. No inhibition of MMP-9 and MMP-2 were found in both LPS-activated astrocytes and microglia.

**Conclusions:**

The present in vitro study suggests that CCR5 antagonist compounds, through their ability to inhibit MMP-9 expression and levels, might have a great potential for the treatment of HIV-associated neurologic damage.

## Introduction

The widespread use of antiretroviral drugs has dramatically modified the natural history of HIV infection, causing a significant reduction in the HIV-associated mortality and morbidity. However, despite complete virologic suppression under treatment, many HIV-infected patients exhibit a persistent state of immune activation which leads to the development of a variety of inflammatory and metabolic pathologies (atherosclerosis, diabetes, cancer, cirrhosis, neurocognitive disorders, metabolic abnormalities) having a negative impact on the clinical progression of HIV infection [1]. To prevent the onset of AIDS and non-AIDS related comorbidities, it is crucial to develop strategies of intervention capable of blocking HIV replication and down-regulating the state of chronic inflammation.

The CCR5 antagonists are a new class of antiretroviral drugs used for the treatment of patients infected with R5-tropic virus [2], [3]. Recent lines of evidence suggest that maraviroc (MVC), the only CCR5 antagonist approved for clinical use, has beneficial immunologic effects beyond its capacity to inhibit virus entry [4]. This drug might have a potential role in the downregulation of HIV-associated chronic inflammation by blocking the recirculation and trafficking of leukocytes within the inflamed tissues.

Leukocyte trafficking and local inflammation plays a prominent role in the physiopathology of HIV infection of the central nervous system (CNS) [5]. Indeed, the development of HIV-associated neurological disorders is associated with increased migration of leukocytes into the CNS, which can disrupt the blood-brain barrier (BBB) and propagate neuroinflammation. These pathologic processes result in BBB permeability, glial activation, and neuronal compromise, all of which contribute to CNS damage [6]. The entry of leukocytes into the CNS is dependent in several factors including the expression of matrix metalloproteinases (MMP) [7].

MMPs are a large family of zinc-containing endopeptidases that degrade extracellular matrix and basement membrane compounds (collagens, gelatin, laminin, fibronectin) [8]. They share common structural domains, but differ with respect to their cellular sources, inducibility and efficiency of substrate utilization. The expression and the activity of MMPs are tightly regulated. Most of the regulatory mechanisms mediated by soluble factors occur primarily at the transcriptional level and involve phosphorilaton of serine-threonine kinases related to the mitogen-activated proteine kinase (MAPK) superfamily [9]. Posttranscriptional regulation of MMPs includes their secretion as latent enzymes and proenzyme activation in the extracellular milieu by different proteinases. Inhibition of MMP activation and proteolytic activity in the extracellular milieu is controlled by a unique family of natural tissue inhibitors (TIMPs) which form with active MMPs stable, non-covalent enzyme-inhibitor complexes [10].

There is evidence that an altered production and secretion of MMPs and TIMPs might be involved in the development of HIV-associated brain injury, contributing to breakdown of the BBB, spreading of HIV into CNS, degradation of myelin and induction of neuronal death [11], [12]. Brain macrophages, microglia and astrocytes represent the candidate cells for the production of MMP within the CNS.

In previous studies, we demonstrated that antiretroviral drugs inhibit MMP-2 and MMP-9 secretion and expression in LPS-stimulated astrocytes and microglia [13]. More recent data shows that antiretroviral compounds can also significantly downregulate the expression of MMP-9 in HIV-naturally infected mononuclear cells [14], indicating that antiretroviral drugs may mitigate MMP-mediated damage by mechanisms that are independent from their ability to block HIV replication. These findings may have great therapeutic implications, suggesting that MMPs may represent a potential target for the development of adjunctive strategies for the management of HIV infection, especially within CNS [12].

On the basis of these observations, since the release of MMPs from glial cells may represent a key event in the pathogenesis of HIV-associated demyelination, we investigated whether CCR5 antagonist MVC was able to modulate the activity and/or the expression of gelatinases A (MMP-2) and B (MMP-9) released from glial cells. Our results indicated that MVC is able to dose-dependently inhibit the release and the expression of MMP-9 from PMA-activated astrocyte cell cultures.

## Materials and Methods

### Reagents

Dulbecco's modified Eagle's medium (DMEM), fetal bovine serum (FBS), penicillin and streptomycin were obtained from GIBCO (Paisley, Scotland). Gelatin, DNase 1, poly-L-lysine (PLL), trypsin, lipopolysaccharide (LPS), 1,10 phenanthroline (PA), Trypan Blue, Thiazolyl Blue Tetrazolium Bromide (MTT) were provided by Sigma (St. Louis, MO, USA.). Maraviroc (MVC) (Celsentri) was from Pfizer, Inc. (New York, NY, USA). Standard proteins and R-250 Coomassie Brilliant Blue were purchased from Bio-Rad (Hercules, CA, USA).

Anti glial fibrillary acidic protein (GFAP) antibodies were purchased from Serotec (Oxford, UK). Purified MMP-2 and MMP-9 were purchased from Alexis Biochemicals (San Diego, CA, USA). The MMP-9 was derived from human neutrophil granulocytes and thus also contained covalent MMP-9-NGAL complex. Primer pairs specific for MMP-2, MMP-9 and GAPDH were from Sigma Genosys (Cambridge, UK). RNeasy mini kit and QuantiTect Reverse Transcription was from Qiagen (Valencia, CA, USA).

### Cell cultures

#### Ethics Statement

All experimental procedures involving animals were carried out in accordance with the NIH Guide for the Care and Use of Laboratory Animals and approved by the Institutional Animal Care and Use Committee of University of Bari, Italy. All efforts were made to minimize the number of animals used and to ameliorate their suffering.

#### Preparation of microglial and astrocyte cultures

Microglial cells were prepared from primary cell cultures of neocortical tissues from 1-day-old rats as described by Nakajima et al. [15]. Briefly, brains were cleaned of meninges and blood vessels, and the dissected neocortical tissues were minced by passage through a stainless steel mesh (40 mesh) and incubated with 0.25% trypsin and 0.01% DNase in DMEM for 10 min at 37°C. After addition of FBS, the dissociated cells were passed through a 100 mesh and viability of cells was assessed by Trypan Blue dye exclusion. Cells were plated in PLL-coated flasks (75 cm^2^) at a density of 1.5×10^7^ viable cells/flask in DMEM, 100 U/ml penicillin, 100 µg/ml streptomycin, 10% FBS and maintained at 37°C in a 5% carbon dioxide incubator with a renewal of medium twice a week. After 7–10 days in culture, the flasks were shaken for 10 min at 100 r.p.m. on a rotary shaker and the cells floating into the medium were transferred to plastic dishes and allowed to adhere at 37°C. Unattached cells were removed after 1 h by washing with DMEM. The strongly adhering cells showed a homogeneous morphology characteristic of microglia as reported by several authors [16], [17]. By immunocytochemical analysis, >98% of the cells stained positively with anti-mouse monocyte/macrophage antibody and were negative with anti-GFAP antibodies.

For the preparation of astrocyte cultures, oligodendrocytes were separated from the astrocytes by mechanical dislodging and then the astrocytes were obtained by trypsinization (0.25% trypsin/0.02% EDTA) [17]. Astrocytes were purified by three repetitions of replating and trypsinization to deplete cultures of microglia and oligodendrocytes. The purity of the final cell culture was assessed by immuno-staining for GFAP. More than 98% of the cells were GFAP-positive in all the preparations.

#### In vitro MVC treatment of microglia and astrocytes activated with LPS or PMA

One day after seeding at a density of 1×10^5^ cells/ml in 96 well-plates, the microglial cells and astrocytes were washed once serum-free DMEM, activated with LPS at the final concentrations of 10 µg/ml or with PMA 100 nM and simultaneously treated with MVC at the final concentrations of 0.2 nM, 2 nM, 20 nM and 200 nM. Negative and positive control supernatants were obtained from unstimulated and untreated microglial cells and astrocytes in serum-free DMEM (negative control), cells stimulated with LPS or PMA (positive control). The incubations were performed in a final volume of 100 µl of serum-free DMEM for 24 h at 37°C, 5% carbon dioxide (CO_2_). At the end of the incubation period, the medium was collected, centrifuged at 2100 rpm and cell culture supernatants stored at −20°C until use. The cells were subjected to the test of cell viability by MTT or lysed for RNA isolation.

### MTT cell viability assay

Cytotoxicity of astrocyte and microglial cells after LPS or PMA-activation and treatment with MVC was detected using MTT [3-(4,5-dimethylthiazol-2-yl)-2,5-diphenyl tetrazolium bromide] assay [18]. This assay is based on the reduction of MTT by the mitochondrial succinate dehydrogenase in viable cells, to a blue formazan product which can be measured spectrophotometrically using a microplate reader.

Briefly, after treatment for 24 h with MVC the culture medium was removed and cells were rinsed in PBS and incubated at 37°C, 5% CO_2_ for 2 h with 0.5 mg/mL MTT. Reaction was stopped by removing the medium and the formazan crystals in the cells were solubilized with absolute ethanol. The absorbances at 560 and 690 nm were determinated by a VersaMax Microplate Reader (Molecular Devices, Sunnyvale, CA, USA). The difference between the absorbance of each sample at 560 and 690 nm was measured. The value of the untreated sample (control) was set at 100% and the cell viability was expressed as percentage of control.

### Detection of gelatinases

Gelatinases in cell culture supernatants were determined by sodium dodecylsulphate-polyacrylamide gel electrophoresis (SDS-PAGE) zymography according to a modification of the method of [19] as described by [13]. Briefly, 50 µl of culture supernatant was supplemented with 30 µl of electrophoresis- loading buffer containing SDS. The samples were then separated in a 7.5% polyacrylamide gel which had been copolymerized with 0.1% (w/v) gelatin. Stacking gels contained 5.4% polyacrylamide. The electrophoresis was performed at 4°C for ∼18 h at 80 V. After electrophoresis, the gels were washed for 2×20 min in 2.5% (w/v) Triton X-100/10 mM CaCl_2_ in 50 mM Tris-HCl, pH 7.4 (washing buffer) in order to remove SDS, and then incubated for 24 h at 37°C in 1% (w/v) Triton X-100/50 mM Tris-HCl/10 mM CaCl_2_, pH 7.4 (developing buffer). For the development of the enzyme activity, the gels were stained with Coomassie Brilliant Blue R-250 and destained in methanol/acetic acid/water. Gelatinase activity was detected as a white band on a blue background and was quantified by computerized image analysis through two-dimensional scanning densitometry using the Image Master 1D program (Pharmacia Biotech, Uppsala, Sweden). Gelatinase activity was expressed as optical density (OD)×mm^2^, representing the scanning area under the curves which takes into account both brightness and width of the substrate lysis zone.

### Reverse Transcription-polymerase chain reaction (RT-PCR)

Total cellular RNA was extracted from astrocyte cells using the Qiagen RNeasy mini kit according to the manufacturer's instructions. The concentration and purity of the isolated RNA were determined by using the NanoDrop 1000 spectrophotometer (Thermo scientific).

Complementary DNA (cDNA) was synthesized from 500 ng RNA by using the QuantiTect Reverse Transcription kit according to manifacturer's instructions. A total of 1 µl of reverse transcription products was used to amplify a 591 bp fragment using specific primers (sense 5′-GTC ACT CCG CTG CGC TTT TCT CG-3′; antisense 5′-GAC ACA TGG GGC ACC TTC TGA-3′) for the rat MMP-2 sequence and a 541 bp fragment using specific primers (sense 5′-CGG AGC ACG GGG ACG GGT ATC 3′; antisense 5′-AAG ACG AAG GGG AAG ACG CAC ATC 3′) for the rat MMP-9 sequence. Amplification of a 308 bp fragment of rat GAPDH (sense 5′-TCC CTC AAG ATT GTC AGC AA-3′; antisense 5′-AGA TCC ACA ACG GAT ACA TT-3′), a relatively invariant internal reference RNA, was performed in parallel. Twenty-five cycles of PCR were carried out, each one consisting of denaturation at 94°C, annealing at 59°C and extension at 72°C in a thermal cycler (PTC- 100 Programmable Thermac Controller, MJ Research, Inc., Walthan, MA, USA). PCR products were visualized by ethidium bromide staining in 1.5% agarose gels. Gels were then processed for densitometric analysis as described for protein gels. Amplification of the target cDNAs were normalized to GAPDH expression.

### Statistical analysis

Data from at least three different experiments were used for statistical analysis. Original data were converted into %-values of positive control and mean± SD was calculated. Values were compared using one-way analysis of variance (ANOVA) followed by the post-hoc Student Newman-Keuls test (multiple comparison).

## Results

### Maraviroc reduces MMP-9 levels in PMA-stimulated astrocytes but not in LPS-stimulated astrocytes

**Figure 1:**
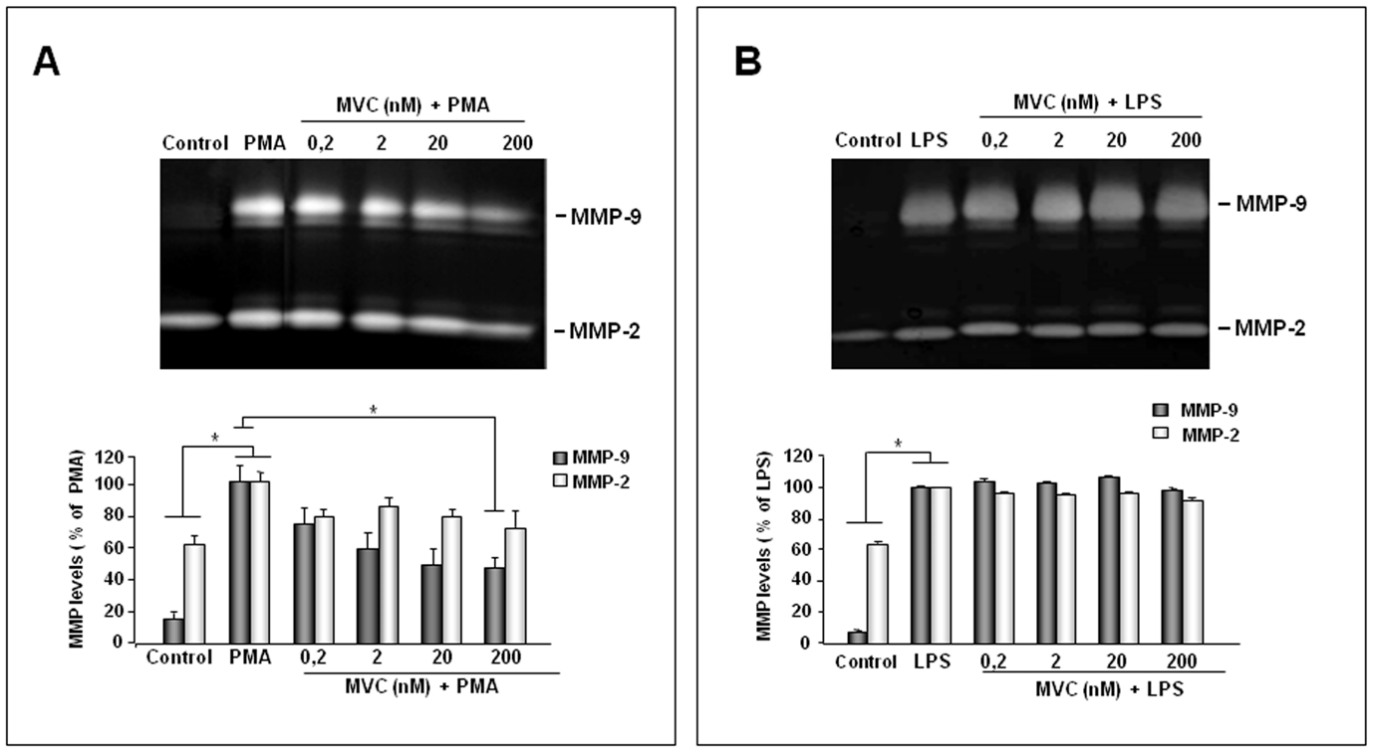
Effect of MVC treatment on MMP-2 and MMP-9 levels in astrocyte culture supernatants. Primary astrocytes (1×10^5^ cells/ml), incubated in serum-free DMEM, were treated with MVC at the indicated concentrations in the presence of PMA (100 nM) (A) or LPS (10 µg/ml) (B). Negative and positive control supernatants were obtained from unstimulated and untreated astrocytes in serum-free DMEM (control) and cells stimulated with LPS or PMA, respectively. After 24 h of incubation equal amounts of cell culture supernatants were subjected to gelatine zymography as described in the Methods section. Representative gels are reported in (A) and (B). MMP-2 and MMP-9 were identified by their apparent molecular mass of 67 and 92 kDa, respectively, using pre-stained molecular weight markers (Bio-Rad). Histograms represent results, expressed as mean±SD, after scanning densitometry and computerized analysis of gels, from at least three independent experiments with different cell populations. Asterisk represents values statistically different from positive control (PMA-treated astrocytes) (One-way Anova followed by Student-Newman-Keuls post hoc test; * = p<0.05).

Primary astrocytes were stimulated with PMA or with LPS and simultaneously treated with different concentrations of MVC in serum-free DMEM. Cell supernatants collected after 24 h incubation were subjected to gelatin zymography for the assessment of MMP-2 and MMP-9 levels. As shown in the representative gels in Fig. 1, cell culture supernatants from untreated and unstimulated astrocytes (control) secreted only the 67 kDa MMP-2 and traces of the 92 kDa MMP-9. LPS or PMA-activation of astrocytes significantly induced MMP-9 as well as MMP-2 levels when compared with those detected in negative controls. The *in vitro* treatment with MVC dose-dependently inhibited levels of MMP-2 and MMP-9 in PMA-activated astrocytes (Fig. 1A), whereas it has no effect on both MMP-9 and MMP-2 levels in LPS-activated astrocytes (Fig. 1B). The quantitation of the data from at least three experiments with different cell populations indicated that the treatment with MVC at the highest concentration of 200 nM induced in PMA-activated astrocytes a statistically significant inhibition of MMP-9 (Fig. 1A). Concerning MMP-2, although the treatment of PMA-activated astrocytes with MVC reduced MMP-2 to levels found in control conditions, the statistical analysis of data indicated that this inhibition is not significant.

**Figure 2:**
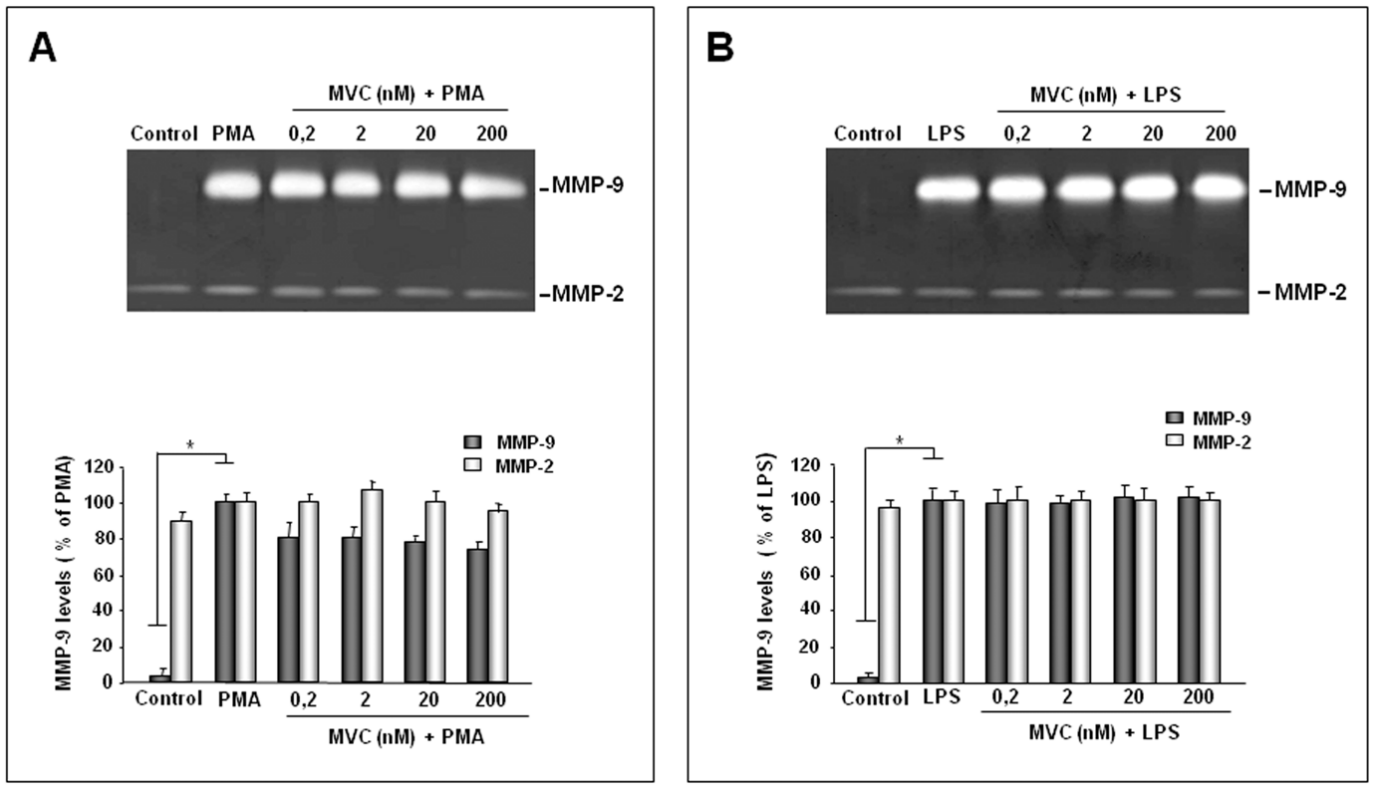
Effect of MVC treatment on MMP-2 and MMP-9 levels in microglial culture supernatants. Primary microglial cells (1×10^5^ cells/ml), incubated in serum-free DMEM, were treated with MVC at the indicated concentrations in the presence of PMA (100 nM) or LPS (10 µg/ml). Negative and positive control supernatants were obtained from unstimulated and untreated microglia in serum-free DMEM (control) and cells stimulated with LPS or PMA, respectively. After 24 h of incubation equal amounts of cell culture supernatants were subjected to gelatine zymography as described in the Methods section. Representative gels in A and B show MMP-2 and MMP-9, as identified by their apparent molecular mass of 67 and 92 kDa, respectively, using pre-stained molecular weight markers (Bio Rad). Histograms represent results, expressed as mean±SD, after scanning densitometry and computerized analysis of gels, from at least three independent experiments with different cell populations. No statistically significant inhibition of MMP-2 and MMP-9 in both PMA- and LPS-activated cells was observed. (One-way Anova followed by Student-Newman-Keuls post hoc test).

The analysis of the effect of MVC on microglial cells showed no inhibition of MMP-2 in both PMA- and LPS-activated cells. With regard to MMP-9, a 25% decrease of MMP-9 levels was observed in PMA-activated microglia (Fig. 2), although this inhibition was not statistically significant.

### Cytotoxicity of astrocytes after treatment with maraviroc

**Figure 3:**
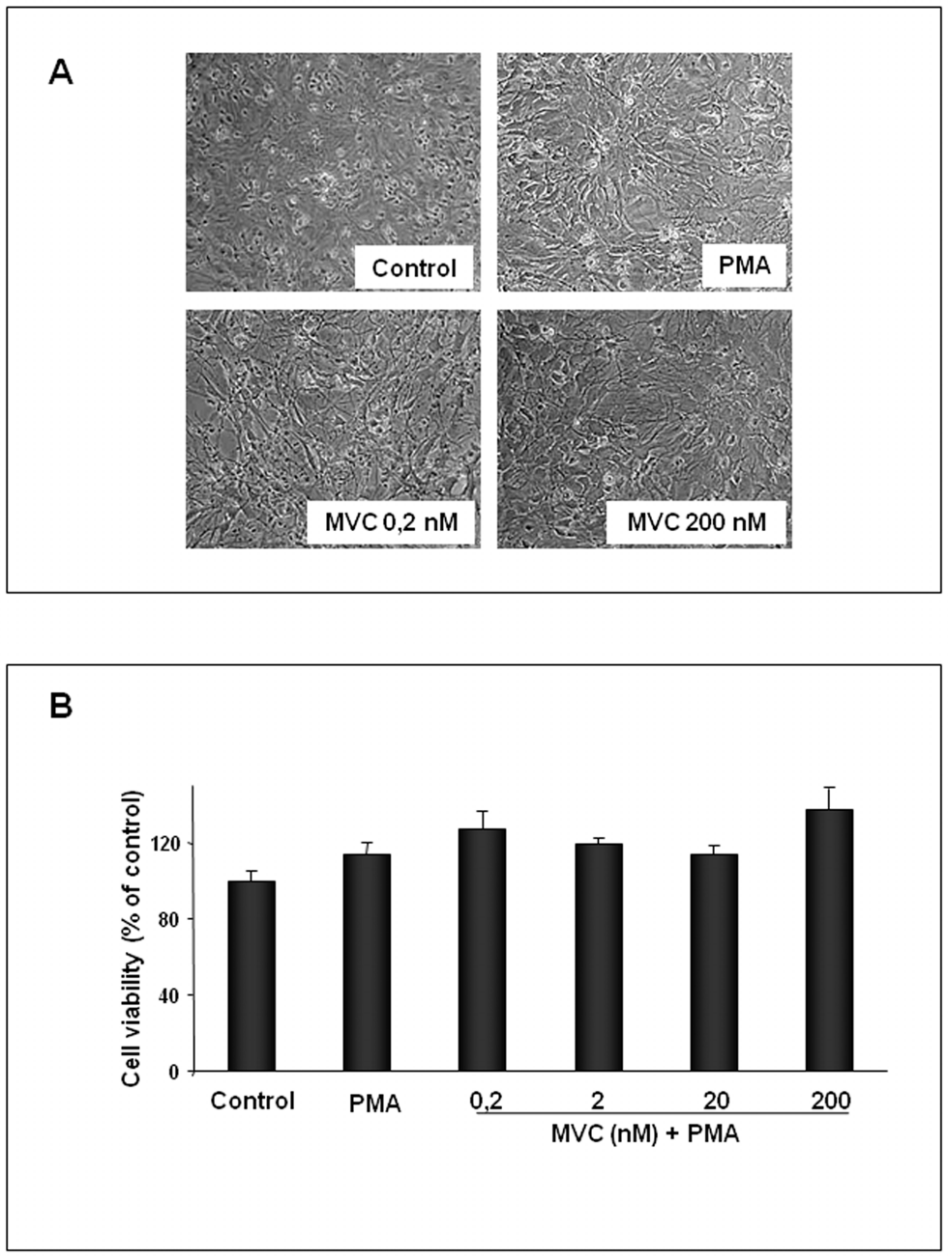
Effect of MVC on astrocyte morphological changes and cell viability. Primary astrocytes (3×10^4^), were activated with PMA 100 nM and simultaneously treated with different concentrations of MVC for 24 h at 37°C, 5%CO_2_ in serum-free DMEM (Control). (A) Photomicrographs show representative results of three independent experiments. Cell morphology was observed under phase-contrast microscope (Magnification 50×). (B) Cell viability was examined by MTT assay. The results are expressed as percentage of surviving cells over control cells. Data are presented as mean ± SD for three separate experiments with independent cell populations.

As shown in Fig. 3A in the absence of stimulation cultured rat astrocytes exhibited a flattened, polygonal morphology, typical of unstimulated astrocytes. The activation of astrocytes with PMA induced a change in a process-bearing stellate morphology, characteristic of reactive astrocytes. This latter morphology was displayed also by PMA-activated astrocytes treated with the lowest concentration of MVC. By contrast, when PMA-activated astrocytes were treated with 200 nM MVC, cell morphology did not change compared to that of control cells.

As assessed by the microscopical observation (Fig. 3A) and the MTT assay (Fig. 3B) we did not observe any significant variation in the number of astrocytes between PMA-stimulated and MVC treated cells, indicating that at all concentrations used in this study, MVC alone or together with PMA did not show cytotoxicity to astrocyte cells.

### Maraviroc does not directly inhibit the activity of purified MMP-2 and MMP-9

**Figure 4:**
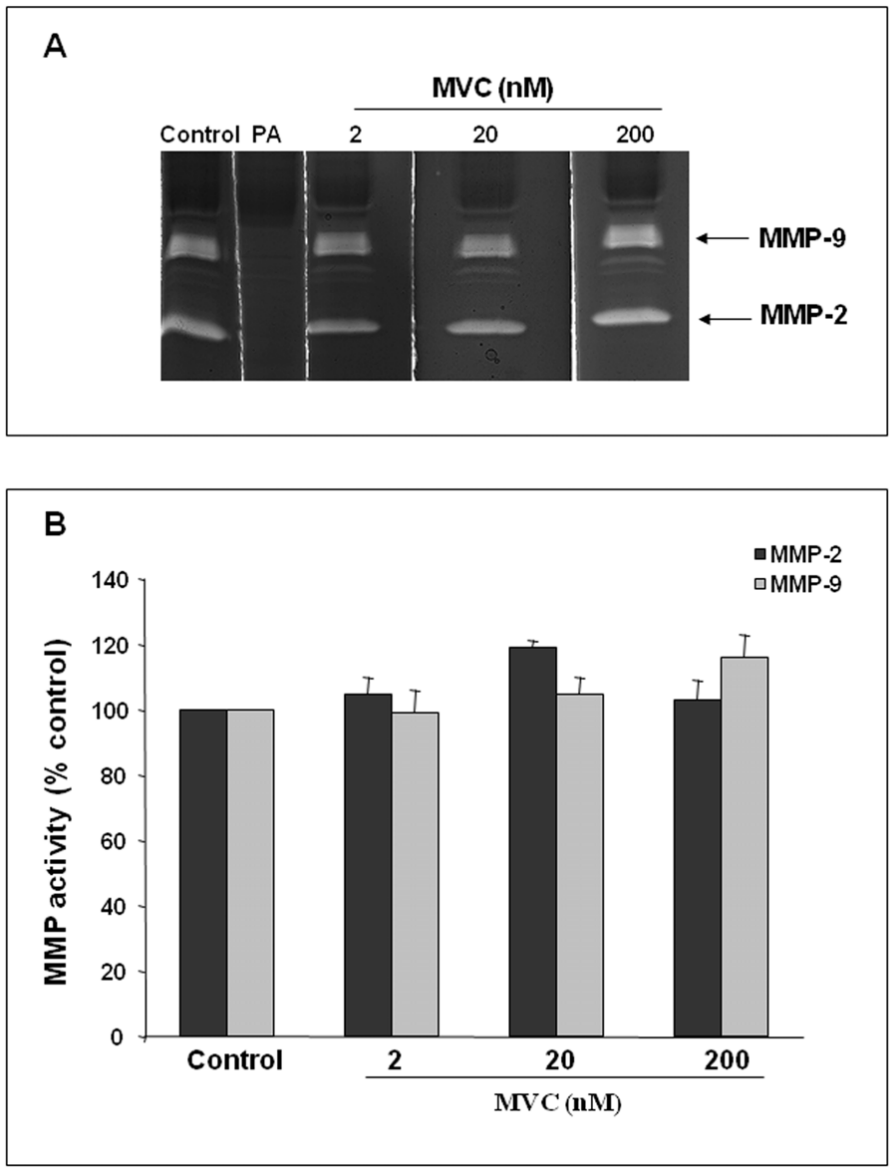
Zymographic analysis of MMP-2 and MMP-9 activity after *in vitro* incubation with MVC. Purified standard MMP-2 (0.35 ng) and MMP-9 (0.88 ng) were subjected to gelatin zymography as described in the text. After the electrophoresis, the gel was cut into lanes and each lane was incubated in the absence (control) or in the presence with MVC at the reported concentrations during the development of the zymograms. 1,10 phenantroline (4 mM) (PA) was used as a positive control. A: Staining and destaining of the gels revealed that inhibition of both MMP-2 and MMP-9 activity was observed only in the lane incubated in the presence of PA, but not in the lanes developed in the presence of MVC. B: Percentage of MMP-9 and MMP-2 inhibition of repeated analyses calculated in comparison to control.

In an in vitro assay, we also tested the ability of MVC to inhibit MMP-2 and MMP-9. To do this, 0.88 ng of purified MMP-9 and 0.35 ng of purified MMP-2 were separated by gelatin zymography and the zymogram was incubated in developing buffer containing MVC at the final concentrations of 2 nM, 20 nM and 200 nM. As a control, MMP-2 and MMP-9 were incubated in the presence of 4 mM 1,10 phenantrolin (PA), an inhibitor of MMPs. As showed in Fig. 4, PA treatment completely blocked the activity of both MMP-2 and MMP-9, whereas MVC did not exert any direct inhibition on the enzymatic activity of MMP-2 and MMP-9.

### Inhibition of MMP-2 and MMP-9 mRNA in astrocytes by maraviroc

**Figure 5:**
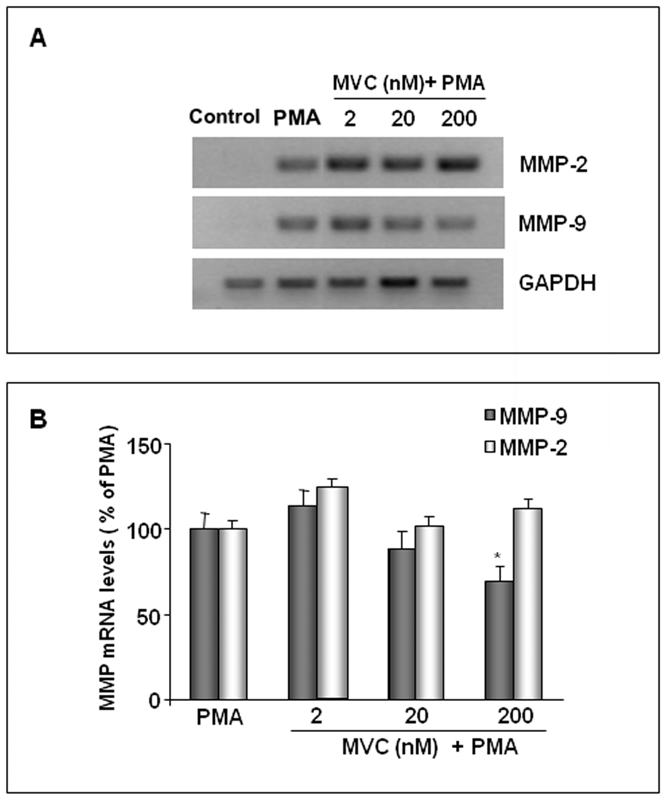
Inhibition of MMP-9 mRNA expression in PMA-activated astrocytes by MVC. Primary astrocytes (1×10^5^ cells/ml), incubated in serum-free DMEM (Control), were activated with 100 nM PMA (positive control) and simoultaneusly treated with MVC at the indicated concentrations. The isolated RNA samples were analyzed by RT-PCR, using the primer pairs specific for MMP-2, MMP-9 and GAPDH. The products were run on a 1,5% agarose gel containing ethidium bromide. The bands were visualized under UV. Representative results are shown in A. Quantitation of the above experiment and two others after scanning densitometry are shown in B. Positive control MMP-2 and MMP-9 mRNA were set at 100%, and the treatments with MVC represented as percent of positive control (mean± SD). Asterisk indicates statistically significant decrease in comparison to positive control. (One-way Anova followed by Student-Newman-Keuls post hoc test; * = p<0,05).

To determine whether the inhibition of MMP-9 levels by MVC in PMA-stimulated astrocytes results from the inhibition of mRNA, RT-PCR was performed. As shown in Fig. 5A, MVC dose-dependently inhibited the PMA-induced expression of MMP-9 mRNA, whereas the expression of MMP-2 and GAPDH mRNA, a housekeeping gene product used as an internal control, was unchanged. Quantitation of results from independent experiments with different cell populations, after normalization with GAPDH, showed a statistical significant inhibition of MMP-9 expression in cells treated with the highest concentration of MVC (Fig. 5B). The RT-PCR of microglial cells gave results which were consistent with the data observed by zymography (data not shown).

## Discussion

Despite the success of antiretroviral therapy (ART) in controlling HIV replication and disease progression, HIV-associated neurological disorders remain an important clinical challenge that still affect nearly 50% of HIV-infected individuals [20], [21]. In particular, several data suggest that HIV may continue to affect the brain even in the presence of effective ART [22], [23]. The large majority of brain-derived HIV strains use mainly CCR5 coreceptor for gaining access into the CNS. It is known that CCR5 has been involved in lymphocyte and mononuclear phagocyte recruitment in the CNS of various neuroinflammatory diseases, including HIV infection [24], [25]. Therefore, particular attention has been dedicated to drugs able to target selectively the CCR5 coreceptor, such as CCR5 antagonists.

MVC is the first CCR5 antagonist approved in clinical practice for the management of HIV infection. Since this drug has been recently introduced into clinical practice, most of its extravirological effects on other factors which are possible causes of development of neurological syndromes remain to be clarified.

In this paper, we studied the effects induced by MVC on the synthesis and the release of MMPs by glial cells and we showed evidence of the ability of this drug to inhibit the release and the expression of MMP-9 and, to a lesser extent of MMP-2, in PMA-activated astrocytes. A slight but not significant inhibition of MMP-9 was also seen in PMA-activated microglial cells after in vitro MVC treatment.

HIV infection of host cells has been shown to trigger the expression of MMPs, in particular MMP-9 [26], [27]. The increased levels of MMP-9 by leukocytes facilitate their entry into CNS. This process disrupts the basement membrane that surrounds the vasculature, resulting in blood–brain barrier (BBB) impairment. Within the CNS parenchyma, brain macrophages, microglia and astrocytes not only replicate the virus actively but also play an important role in HIV-associated neural injury through the production of inflammatory mediators and various neurotoxins, including MMPs [28]. In the CNS, high MMP content and its indiscriminate localization results in perpetuation of an inflammatory response, which contributes to demyelination and neuronal or oligodendrocyte death. In the light of these considerations, MMPs can be considered as an important target for an innovative therapeutic approach in alternative or in addition to the pharmacological therapy, especially for HIV-associated neurologic disorders for which we have no specific therapies so far. In previous studies, we showed that antiretroviral drugs, particularly HIV protease inhibitors, are capable of inhibiting MMP-2 and MMP-9 secretion and expression in LPS-stimulated astrocytes and microglia [13]. More recent data indicate that antiretroviral compounds can also significantly downregulate the expression of MMP-9 in mononuclear cells naturally infected with HIV [14]. These findings could have great therapeutic implications, suggesting that antiretroviral drugs might counteract MMP-mediated damage in the brain by mechanisms that are independent from their capacity to suppress HIV replication. On the other hand, the ability of antiretroviral drugs, especially protease inhibitors, to exert extravirologic effects at various cellular patways has been previously demonstrated [29], [30].

MVC through the ability of downregulate MMP expression in rat astrocytes might contribute to counteract the chronic activation of glial cells into the CNS, thus limiting the progression of demyelination and neurodegeneration associated with HIV infection. In this respect, the morphological changes observed in MVC-treated astrocytes compared with those activated with PMA, may suggest that at the cellular level MVC may act by counteracting the activation of astrocytes.

To explain the reduced effect of MVC on MMP-9 levels in microglial cells, we detected the expression levels of CCR5 in astrocytes and microglia by using real-time PCR. As a result, we found similar levels of CCR5 on both cell populations in basal conditions as well as in PMA-activated cells, suggesting that that the different effect of MVC on the two cell populations do no depend from levels of CCR5 (data not shown). A possible explanation for the reduced inhibitory effect of MVC on MMP-9 in microglial cells may arise from the observation that microglia are the most reactive cell population of brain parenchyma. Microglia rapidly transform from a resting to an activated morphology in response to different inducers such as LPS, PMA, cytokines and chemokines. The products of activated microglia are pro-inflammatory cytokines, chemokines, nitric oxide, superoxide radicals which, in turn, may induce the production of MMP-9. Therefore, the induction of MMP-9 in activated microglial cells may be due to the cooperative interaction of multiple signaling pathways suggesting that the functional interplay of these pathways may in part counteracts the inhibitory effect of MVC.

Recent findings suggest that CCR5 antagonists could have immunomodulant properties beyond the pure anti-HIV inhibitory activity [31], [32]. By preventing signalling through the CCR5 receptor, CCR5 antagonists may downregulate immune activation, T-cell apoptosis and cytokine expression. In a recent study, we provided evidence that that PBMCs from HIV-infected patients exhibited diminished chemotactic migration following in vitro and ex vivo treatment with CCR5 antagonist MVC [33]. Moreover, in a set of in vitro experiments, we demonstrated that MVC was able to downregulate the migration of innate immune cells, such as monocyte/macrophages and dendritic cells [34]. These data findings suggest that CCR5 antagonists might have a potential role in reducing the severity of neuroinflammation by targeting the CCR5-leukocyte recruitment pathway to the CNS. On the other hand, experimental data in a murine model of demyelination showed that an anti-CCL5 monoclonal antibody reduces T-cell and macrophage accumulation within the CNS and improves neurological function [35]. On the basis of these considerations, the use of CCR5 antagonists for the management of HIV brain infection could have a double therapeutic effect: blocking viral entry and counteracting leukocyte recruitment and neuroinflammation.

To study the effect of MVC we activated glial cells with two different immunogens that can stimulate the release of MMP-9 with different mechanisms of action. In particular, LPS interacts with CD14 and, by triggering the cascade of the mitogen-activated protein kinase (MAPK) downstream of Toll-like Receptor-4 (TLR-4), induces the expression of MMP-9 [36]–[38]. The PMA, however, crosses the plasma membrane and induces the expression of MMP-9 through activation of MAPK mediated by protein kinase C (PKC) [39].

In our experiments, we demonstrated the ability of MVC to inhibit the expression of MMP-9 in PMA- but not LPS-activated astrocytes. This result could be explained by considering the mechanism of action of MVC at the cellular level.

MVC is a CCR5 antagonist that, by binding to the chemokine receptor, prevents the conformational change of the gp120 viral protein, which is necessary for viral entry into cells, and inhibits the cascade downstream of the receptor for chemokines, by inactivating PKC. Therefore, MVC may act by inhibiting in part the activation of PKC by PMA, thus downregulating the expression of MMP-9. By contrast, the lack of inhibition of MMP-9 by MVC in LPS-activated, could be explained considering that the signal transduction pathway activated by LPS could occur with a PKC-independent mechanism.

This hypothesis that MVC interferes with the activation of MMP-9 at level of gene transcription is also confirmed from the experimental observation that this drug inhibits the expression but not the activity of MMP-9, as demonstrated in our experiments with RT-PCR and in vitro zymography. On the other hand, we can exclude that the reduction of MMP-9 levels and expression, observed in PMA-activated astrocytes by MVC, may be due to cytotoxic effects of this drug. In fact, both the microscopic observation and the cytotoxicity test demonstrate that there are no variations in the number of cells. The present in vitro study suggests that CCR5 antagonist compounds might have a great potential for the treatment of HIV-associated neurological damage. In particular the beneficial effect of MVC could be ascribed to three mechanisms of action: i) inhibition of HIV entry by blocking the interaction between HIV and the chemokine receptor CCR5, that is necessary for the virus to enter host cells.; ii) downregulation of migration of HIV-producing cells across BBB as consequence of CCR5 inhibition; iii) direct inhibition of MMP levels in astrocytes with a mechanism that is independent from its antiviral activity. These findings might have clinical implications on the impact of CCR5 antagonists in neurological diseases associated with MMPs involvement.
